# Fully Mechanically Controlled Automated Electron Microscopic Tomography

**DOI:** 10.1038/srep29231

**Published:** 2016-07-11

**Authors:** Jinxin Liu, Hongchang Li, Lei Zhang, Matthew Rames, Meng Zhang, Yadong Yu, Bo Peng, César Díaz Celis, April Xu, Qin Zou, Xu Yang, Xuefeng Chen, Gang Ren

**Affiliations:** 1 The Molecular Foundry, Lawrence Berkeley National Laboratory, Berkeley, CA 94720, USA; 2 State Key Laboratory for Manufacturing System Engineering, Xi’an Jiaotong University, Xi’an, 710049, P.R. China; 3 School of Electrical Engineering, Xi’an Jiaotong University, Xi’an, 710049, P.R. China; 4 Howard Hughes Medical Institute, University of California, Berkeley, Berkeley, USA; 5 Pfizer BioTherapeutics Pharmaceutical Sciences, 401 N Middletown Rd, Pear River, NY 10956, USA; 6 Pfizer BioTherapeutics Pharmaceutical Sciences, 700 Chesterfield Parkway West, St Louis, MO 63017, USA

## Abstract

Knowledge of three-dimensional (3D) structures of each individual particles of asymmetric and flexible proteins is essential in understanding those proteins’ functions; but their structures are difficult to determine. Electron tomography (ET) provides a tool for imaging a single and unique biological object from a series of tilted angles, but it is challenging to image a single protein for three-dimensional (3D) reconstruction due to the imperfect mechanical control capability of the specimen goniometer under both a medium to high magnification (approximately 50,000–160,000×) and an optimized beam coherence condition. Here, we report a fully mechanical control method for automating ET data acquisition without using beam tilt/shift processes. This method could reduce the accumulation of beam tilt/shift that used to compensate the error from the mechanical control, but downgraded the beam coherence. Our method was developed by minimizing the error of the target object center during the tilting process through a closed-loop proportional-integral (PI) control algorithm. The validations by both negative staining (NS) and cryo-electron microscopy (cryo-EM) suggest that this method has a comparable capability to other ET methods in tracking target proteins while maintaining optimized beam coherence conditions for imaging.

The structural and dynamic characteristics of proteins are essential for understanding their functional activity. The dynamic character of proteins hinders structural determination by conventional approaches, particularly for highly dynamic proteins, such as antibodies, lipoproteins and DNA-protein complexes^1^,^2^. Conventional approaches, such as X-ray and electron microscopy (EM) single-particle reconstruction, require thousands to millions of different molecules to average^3^. Averaging these proteins without prior knowledge of the protein dynamics and fluctuations could potentially fail to detect the dynamic characteristics and blur or eliminate any flexible domains. Therefore, a method to reveal the structure from each single and unique molecule is necessary.

Electron tomography (ET) is a powerful tool to obtain a snapshot of a single-instance biological object from a series of tilted viewing angles. After computerized image alignment and three-dimensional (3D) reconstruction algorithms, a 3D structure can be revealed from a single and individual object, such as a section of a cell^4^, an individual bacterium^4^, large protein complexes^5^ or even a single protein^6^,^7^,^8^. The 3D reconstruction capability of this technique requires a set of high-resolution and high-quality images. However, imaging a target object from a series of tilted angles under high magnification is challenging, especially for imaging proteins. The imperfect mechanical design and control capability often causes ET data acquisition failure due to a significant shift from the targeted imaging area during the tilting process. For example, if an object is 1 μm away from the Eucentric height of the goniometer, the center of this object can shift away by approximately 0.6 μm at the high tilt angle of 60°, which is often larger than the imaging area under a magnification of 100,000×, resulting in a failure to track and image the object.

In the past two decades, several automation-based ET software programs have been developed to control the TEM and allow precise tracking and imaging of a target object and reduce image acquisition time^9^,^10^,^11^,^12^. The early automated method for ET acquisition utilized a pre-illuminated image to calculate the shift from the previous image by cross-correlation and then acquire the real image after compensating for this shift^13^,^14^,^15^. To reduce the overall illumination dose to the target area induced by the pre-illumination step, later methods were developed by introducing a pre-determined tilting trajectory model of the target area^10^,^16^ to predict and correct the shift before image acquisition. Pre-determination of the tilting trajectory of the object is challenging due to the imperfect mechanical design and control of the goniometer, the unevenness of the specimen and environmental vibrations during tilting, which could cause variance in the determined tilting trajectory. An approach to mitigate the influence of those problems has been developed by predicting specimen movements by using nearby tilt angles^17^, which enables dynamic position tracking of the imaging area. However, the imperfect mechanical control capability of the specimen goniometer still requires compensation by electron beam tilting/shifting, particularly under medium to high magnifications (50,000–160,000×). The accumulation of beam tilt/shift processes could lead to a significant residual beam shift, which could degrade the beam coherence and lower the image quality.

Because beam coherence is important for high-resolution imaging, in this study, we propose a method to maintain the optimized beam coherence by only controlling the mechanical stage for tracking and imaging the proteins under medium to high magnification.

## Mechanical control problems

**Figure 1:**
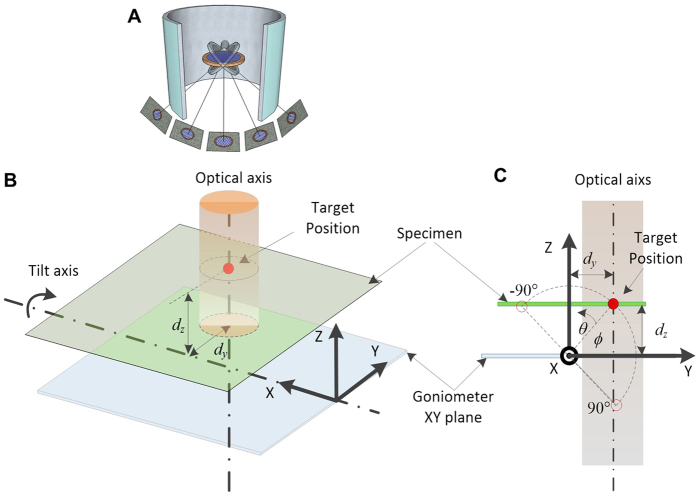
Schematics of the relationship between the optic axis and tilt axis within the TEM. (**A**) A cartoon view of tomography, through a tilted sample stage. (**B**) 3D schematic view of the relationship between the optical axis and tilt axis within the TEM goniometer, specimen and imaging target diagram, where *d*_*z*_ and *d*_*y*_ determine the displacement from the tilt axis to the target position. (**C**) Side view of B, where *θ* = *φ* is the zero-tilt angle of the target position relative to the tilt axis.

The imperfect alignment of the specimen to the Eucentric height, along with imperfections in the manufactured goniometer design, often causes the target area center to shift away from the imaging area during titling (Fig. 1). The major aspects of the imperfect design and mechanical control of the specimen stage by the goniometer can be categorized by the following three phenomena: uneven moving distances (referred to as moving control error), goniometer backlash (backlash error) and an unrepeatable tilt trajectory (trajectory error).

**Figure 2:**
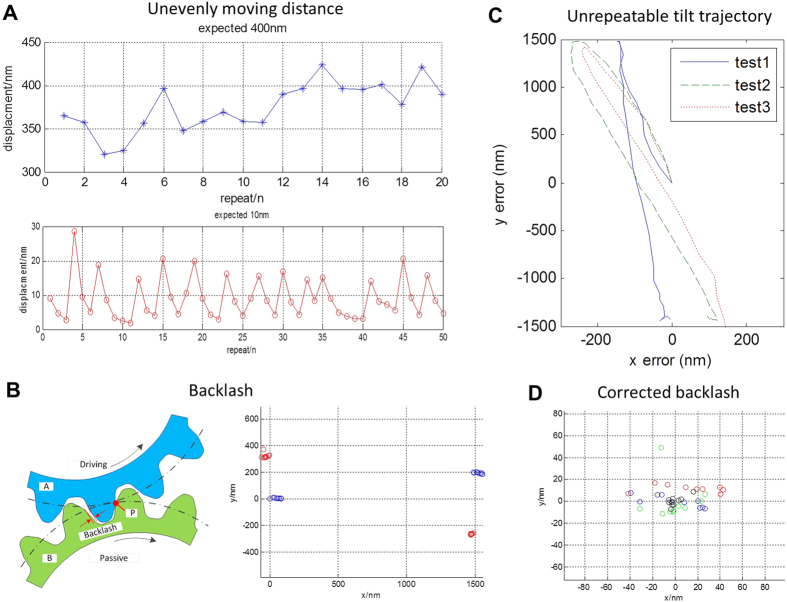
Three major sources of mechanical errors and backlash correction. (**A**) Motion control error from repeated X and Y motions with steps of 400 and 10 nm. (**B**) Backlash error from X and Y motion, caused by uneven gear meshing within the stage driver. (**C**) Trajectory error: unrepeatable tilt trajectory of X and Y resulting from non-ideal mechanical alignment. (**D**) Motion control error after backlash correction. Residual backlash error plot of X and Y motion after backlash elimination.

- Moving control error can be demonstrated by ordering the specimen stage to move a series of identical moving steps, with the actual moving distances measured by cross-correlated images. The results showed that the actual moving distances were uneven and unrepeatable. For example, a continuous movement with a step length of 400 nm showed an actual moving distance in the range of approximately 320–420 nm (Fig. 2A). This moving control error was even worse when a smaller step was used. For instance, a continuous movement with a step length of 10 nm showed that the actual moving distance could range from 2 to 30 nm (Fig. 2A).
- Backlash error is introduced by the backlash of mechanical components and occurs when the goniometer changes its movement direction (Fig. 2B left panel). The backlash error can be as large as approximately 1,500 and 400 nm for X and Y, respectively, on our Zeiss Libra 120 Plus TEM (Fig. 2B right panel).
- Trajectory error may induced by the mechanically imperfect design of the goniometer, environmental vibrations, and misalignments of the tilt axis with the optical axis. For example, by repeating the goniometer tilting three times from −60° to +60° in steps of 1.5°, the trajectories of the same target were neither overlapping nor repeatable (Fig. 2C).

## Backlash elimination

Among the above three major errors, the backlash error is the largest error that is related to the moving direction. Mechanical clearance or lost motion caused by gaps between the gears within the goniometer often generates backlash error (Fig. 2B). The backlash error can typically be significantly reduced by resetting the gear movement direction to the same direction as the previous moving direction. In our program, we move the specimen backward to the targeted moving direction by 5 μm before moving it to the targeted position. This process can reduce the backlash error within a standard deviation for X and Y motions to approximately 27 and 20 nm, respectively (Fig. 2D), which is significantly smaller than the original backlash errors of 1,500 and 400 nm (Fig. 2B).

## Target Position Tracking Control

**Figure 3:**
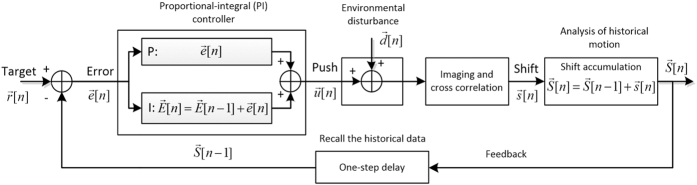
Overview of the Proportional-Integral (PI) control system. Overview of the PI control system. Initially, the PI controller uses the previous errors to calculate the push 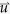
[*n*] for the next goniometer motion to correct the previous positional error. Goniometer motion incurs additional environmental error 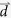
[*n*], which yields the shift 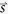
[*n*] through imaging and cross-correlation. The accumulated shift 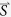
[*n*] is calculated by incorporating the historical motion 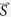
[*n* − *1*] and the current shift 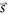
[*n*]. This 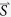
[*n*] is used as historical data for the next tilt loop. The difference of 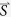
[*n*] from the target 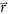
[*n*] is the residual error 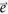
[n]. Within the proportional integral (PI) controller, the accumulated residual error 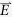
[*n*] is calculated by incorporating the historical error 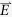
[*n* − *1*] and current residual error 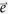
[n].

Tracking of the target object during tilting is still affected by the trajectory error, moving control error, and residual backlash error. The correction of each error is challenging because the errors are convoluted. In our strategy, instead of reducing each error separately, we treated all errors together as an “environmental” disturbance that interrupts the targeting position center during the tilting process. By introducing a closed-loop control system with a proportional-integral (PI) control strategy^18^ (Fig. 3), we significantly suppressed those errors and successfully tracked the target position under 160,000× magnification.

A closed-loop PI control compares the built-up historical data (integral) weighted against the instantaneous error of each step away from a reference point (proportional) to maintain the system (Fig. 3). This system applied to our XY positional tracking is briefly described as follows. For an example of the *n*^*th*^ tilting step, the image shift 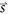
[*n*] is measured by the cross-correlation between the last and current tilting images. The accumulation of all historical image shifts is defined by 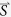
[*n*]. The goal of the control system is to maintain the accumulated shift 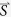
[*n*] as close to the target 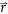
[*n*] as possible at each tilting step (for XY positional tracking, 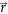
[*n*] is set as zero because a target position of zero corresponds to no positional shift between tilted images, resulting in optimal tracking). However, the actual accumulated shift 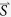
[*n*] is away from the target with a residual error of 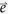
[n], with the accumulation of such residual error defined by 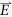
[*n*]. Thus, we apply a goniometer motion 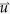
[*n*], which can reduce both the latest residual error 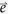
[n] and its accumulated error 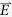
[*n*] (after the 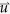
[*n*] motion is complete, we generally wait 10–15 seconds for the stage to stabilize, although this time can be modified). The PI control algorithm works to balance both residual errors via a weight *k*, resulting in the suggested goniometer motion 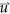
[*n*]. In practice, *k* can be adjusted based on different microscopes and specimen holders to a value between 0 and 1. In every tilt, the achieved image shift 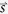
[*n*] is interrupted by an environmental disturbance 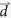
[*n*], which will be controlled in the next loop. This closed-loop system can be described by the following difference equations:


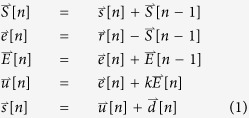


where 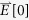 and 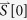 are initialized to 0 and the target 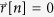 for XY position tracking.

### Measurement of the XY shift of tilted images

**Figure 4:**
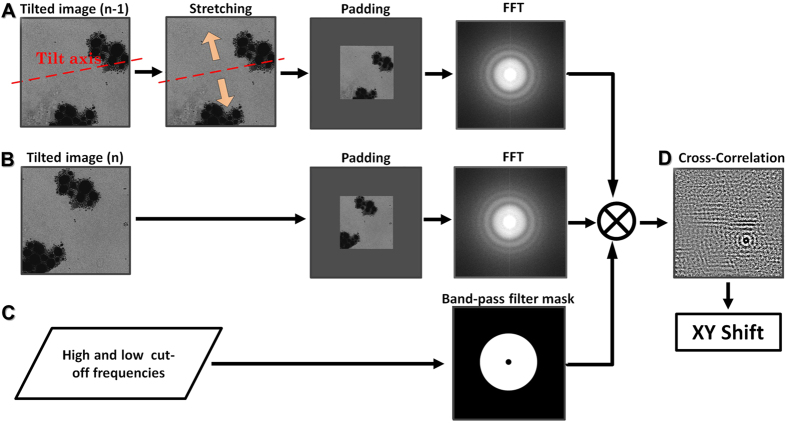
The procedure for measuring the XY shift of tilted images. (**A**) the (n − 1)^th^ tilted image is first stretched along the perpendicular direction to the tilting axis to compensate the effect of the tilt angle difference from the *n*^*th*^ image, and then padded into an area twice as large before Fourier transfer. (**B**) The *n*^*th*^ tilted image was directly padded into an area twice as large for Fourier transfer. (**C**) Based on inputted cut-off frequencies for band-pass filtering, a mask in reciprocal space was generated and applied to calculate the modified cross-correlation. (**D**) The revised Fourier transfer of the modified cross-correlation showed a peak to represent the defined XY shift.

Accurately measuring the XY shift between two sequentially tilted images is critical in this control system. For a robust and precise measurement of the shift, we modified the Fourier space cross-correlation as showed in Fig. 4A and the below equation,


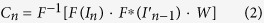


where *C*_*n*_ is the 2D image that represents the distribution of the calculated cross-correlation values (Fig. 4D); *F*^−1^( ) is the inverse Fourier transformation; *F*( ) is Fourier transformation; F^*^( ) is the complex conjugate of *F*( ), defined as the real part of *F*( ) minus the imaginary part; W is the window function used for cut-off the frequencies out of the band-pass range (*i.e.* this would be 1 for within the spatial frequency range of 0.05–0.5, or 0 for outside the range, Fig. 4C); *I*_*n*_ is the image that is twice as large as that of the *n*^*th*^ tilted image, and was padded with the tilted image in the center (Fig. 4B). The *I*’_*n−1*_ is the image that is also twice as large as that of the (*n* − *1*)^*th*^ tilted image, and padded with the (*n* − *1*)^*th*^ tilted image in the center after the tilted image was stretched along its perpendicular direction to the tilting axis to compensate its tilted angle effect from the *n*^*th*^ image (Fig. 4A). The padding process is to increase the image sampling in reciprocal space^19^ and to avoid a potential error for the image with a shift greater than half of the image size. During the above calculation, the spatial envelope was not changed.

In the above band-pass filter process, the cut-off frequencies in a range of 0.05–0.5 was used based on experience. The empirical value could reduce the influence from the cross-correlation of noise and background intensity gradient (resulted by unevenly distribution of ice thickness, negative staining or the electron beam intensity) to the determination of the XY-shift. Although other filter, such as Gaussian type filtering, could provide even precisely value (within ~1 nm), considering the accuracy could be controlled by mechanics is only within ~50 nm, the additional accuracy determined from other fitters would not significant benefit to the XY-shift value feedback to mechanical controlling. Therefore, the simple band-pass filter was used due to self-sufficiency in XY-shift determination.

In the above padding process, we normalized the *n*^*th*^ tilted image and stretched (*n* − *1*)^*th*^ tilted image by shifted their mean values to be “0”, and then added each image to a twice large image that has a flat image value of “0”. It is because the mean values of tilted images are critical for XY-shift determination, since it could influence the quality of the cross correlation. The code used to avoid the affects from the tilted image mean value shown below,

*// measure the shift from img1 to img2*

*number LHC_MeasureShift(image img1, image img2, number &sx, number &sy)*

*{*

*number w,h,w2,h2*

*img1.GetSize(w,h)*

*img2.GetSize(w2,h2)*

*if(w2! = w || h2! = h)*

*{*

*ShowAlert(“Error in function [LHC_MeasureShift]:\nImage sizes were different.”,0)*

*return 0*

*}*

image img1e := NewImage(“”,img1.ImageGetDataType(),w*2,h*2)

*img1e[0,0,w,h] = img1-mean(img1)*

image img2e := NewImage(“”,img2.ImageGetDataType(),w*2,h*2)

*img2e[0,0,w,h] = img2-mean(img2)*

*image img := CrossCorrelate(img1e,img2e)*

*number cc = img.max(sx,sy)*

*sx = w-sx*

*sy = h-sy*

*return cc*

*}*

By using the above method, we seldom encountered algorithm failure during the acquisition on negative stain samples under both medium to high magnification (approximately 50,000–160,000×). However, we did often encounter algorithm failures on cryo-EM samples when imaging a clean ice area. The low contrast of ice only images often provided insufficient signal for tracking XY shift. This cryo-EM success rate could be increased by: i) using home-made lacey carbon film supported grids to prepare the cryo-EM grid; ii) selecting areas that contact narrow boundaries of supporting carbons forming a “star shape” as a center of the targeted area; iii) reducing the magnification to including more carbon film boundaries. Narrow carbon boundaries could provide sufficient signal for calculating XY shift, but would not waste too much space for imaging the vitreous ice and embedded samples.

## Defocus (under focus) tracking control

In addition to positional tracking, defocus (under focus) control, is also important for high-resolution image acquisition during ET tilting, because each tilt must be under a consistent defocus. However, defocus control can become particularly difficult under conditions that include the misalignment of the eucentric height, an imperfectly manufactured goniometer design and mechanical vibrations induced by the XY motion. Moreover, in our instrument, Z-positional mechanical control is convoluted with the Y position, which causes the mechanical controls of the Z position to complicate the XY positional tracking ability. Thus, we introduced a similar PI control system to regulate the beam defocus. The defocus regulating system has four main differences compared with the positional tracking system: i) the variables are scalars rather than vectors; ii) the defocus is defined by CTF fitting utilizing the FFT function within the DM software; iii) the goal of the control system is maintaining the accumulated defocus at each tilting step as close to the desired defocus as possible; and iv) this PI controller is used to change the focus rather than drive the goniometer.

## Measurement of the defocus of a tilted image

**Figure 5:**
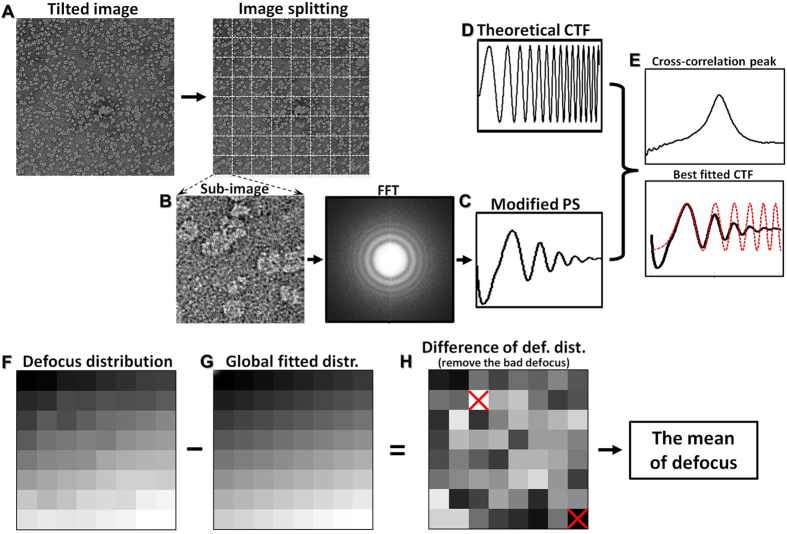
The procedure for defining the defocus of a tilted image. (**A**) The whole micrograph is divided into 8 × 8 mosaic tiles. (**B**) Each sub-image was used to measure the defocus after Fourier transfer. (**C**) Though a modified power spectrum (PS) of the sub-image, (**D**) the curve was compared with the theoretical contrast transfer function (CTF) curve via the calculation of the cross-correlation within a defined frequency range. (**E**) By screening the defocus within a searching range, the defocus which maximized the cross-correlation (CC) between the modified experimental curve and theoretical curve was used as the measured defocus for this sub-image. (**F**) By repeating the above procedure on each of the other sub-images, we obtained the defocus distribution against their whole micrograph. (**G**) The distribution can be fitted with a linear gradient distribution plane. (**H**) By subtracting this plane from the original 2D distribution, we could obtain a distribution of the residuals for defining the “bad” defocus measurements within the sub-images. The bad measured defocus was defined as any residual above twice the standard deviation (such as the two defocuses marked in red crosses). After the bad defocus tiles are removed, the defocus distribution was then re-fitted a linear distribution plane, and the defocus at the center was used as the defocus of the tilted micrograph.

Accurately measuring the defocus of each tilted image is essential for focus control and tracking. The challenge in defining the defocus of a tilted image is the defocus gradient. Our strategy to which defined the tilted image defocus divided the whole micrograph into 8 × 8 mosaic tiles (Fig. 5A), and then defined the defocus of each tile (Fig. 5B).

To define the defocus of each tile, we first Fourier transform the image of a tile, and rotationally average to compute the modified power spectrum *c*_*r*_(*k*) as the following equation,


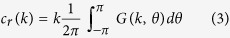


where *k* is the frequency; *θ* is the rotation angle; and *G* is the modulus of the Fourier transfer of the image. Notably, we multiplied *k* during the rotational averaging in order to make the power spectrum isolation center more flat (Fig. 5C). This modification can provide a better comparison to the theoretical contrast transfer function (CTF) (Fig. 5D). The theoretical CTF curve used here was defined as previously reported^20^


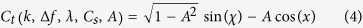



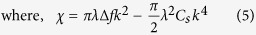


in which, Δ*f* is the defocus (generally *k* and Δ*f* are regarded as independent variable); *C*_*s*_ is the spherical aberration coefficient of the objective lens; *A* is the amplitude contrast (ranging from 0.07 to 0.14 for cryo-samples and 0.19 to 0.35 for negative-stained samples^20^); *λ* is the wave length of the electron.

The modified power spectrum and the theoretical CTF curve are compared ring by ring through computing the correlation coefficient (CC) of each pair of rings as defined below,


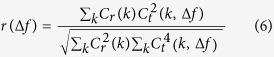


Notably, for images with poor CTF thon rings, only the first several pairs of rings will be used for calculations of the correlation coefficients (Fig. 5J). Since the average correlation coefficient is a unimodal function of defocus, we search the defocus Δ*f* within a given range of defocus (Δ*f*_min_ and Δ*f*_max_) (Fig. 5E). The defocus Δ*f*_*opt*_ should give a maximum CC value and provide the best fit to the experimental curve (Fig. 5E), as defined by the defocus for this particular tile image.

By repeating the above procedure on each of tiles, we obtained the defocus distribution map of the whole micrograph (Fig. 5F). We can calculate the mean of this defocus to represent the defocus of the tilted micrograph. Considering some tiles may be completely wrong due to the low image contrast or the presence of a large dark chunk, we should exclude these bad measurements before calculating the mean of the defocus. To define the tiles with bad defocus, we first fitted the defocus gradient 2D distribution by a linear distribution plane (Fig. 5G), and then subtracted this plane (as average distribution) from the original 2D distribution to obtain a distribution of the residuals. This residual distribution could be used to identify poor defocus defined as that the residual is above twice the standard deviation (Fig. 5H). After tiles with bad defocus were removed, the defocus distribution map of the remaining tiles was fitted again by a linear distribution plane, and the average defocus of this newly fitted plane represented the defocus of this tilted micrograph.

By using the above method, the acquisitions on negative stain samples were seldom encountered failure under both medium to high magnification (approximately 50,000–160,000×). However, for acquisition on cryo-EM samples, the method has difficult to define the defocus on the sub-images of clean ice area that contains little embedded sample. To increase the success rate, we intended to include more supporting carbon film and operated under a relatively lower magnificent, such as 50,000 to 80,000.

## Graphical user interface

The control of the Libra 120 TEM was operated by the protocol of RS232 communications with WinTEM software^21^ from Carl Zeiss SMT Ltd. The controlling scripts were coded in C program. The communication scripts were executed through the interface of Gatan DigitalMicrograph (DM)^22^. For example, a script, *LHC_GetGonPos,* was coded to read the stage positions as following,

*// reading stage position*

*void LHC_GetGonPos(number &x, number &y, number &z, number &t)*

*{*

*string str = “G300xxxxxxxxyyyyyyyyzzzzzzzztttttttt”*

*Leo_Command(“G300”,str)*

x = val(left(str.right(32),8))*1e-9

y = val(left(str.right(24),8))*1e-9

z = val(left(str.right(16),8))*1e-9

t = val(str.right(8))/1e4/180*pi()

*}*

while another example script, *LHC_SetGonX*, was coded to drive the goniometer along the X-axis for xxxxxxxx nm distance as below,

*// driving stage X position*

*void LHC_SetGonX(number x)*

*{*

Leo_Command(“S303” + LHC_Decimal(x*1e9,8))

*}*

where, *Leo_Command* is a user input/output interface command to submit a RS232 protocol to WinTEM for controlling the TEM from the Gatan DM interface. G300 is one of RS232 commands for reading the goniometer positions (X-axis, Y-axis, Z-axis, Tilt-angle, in a format of 8 digitals, *i.e.* xxxxxxxxyyyyyyyyzzzzzzzztttttttt, within a range of −1000000 to +1000000). The units of X, Y, Z positions are nanometer (nm), while the unit of T position is in 1/10000 of a degree. S303 is the RS232 command to drive the goniometer along the X-axis, while the *LHC_Decimal* is a script to convert a decimal value into a format of 8 digitals.

**Figure 6:**
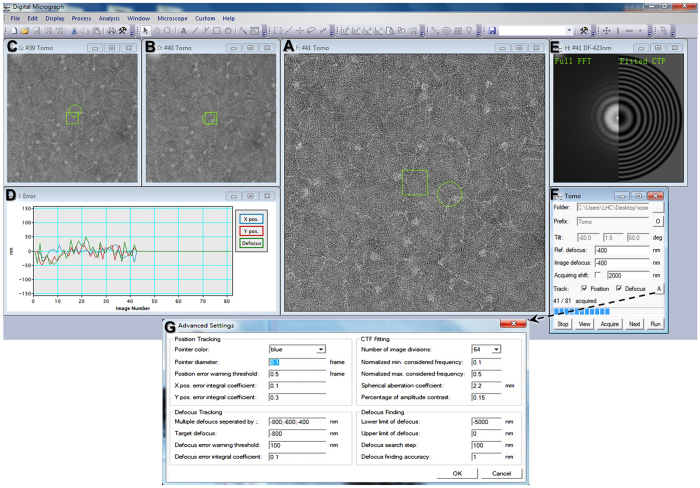
Graphical user interface of the tomography software. (**A**) Imaging area of the current, (**B**,**C**) previous and next previous collected images. (**D**) Plot of the positional and defocus errors during tomography collection. (**E**) Fast-Fourier transformation (FFT) of the current image and fitted contrast transfer function (CTF) curve. (**F**) Control panel for tomographic parameters, options, and process status. (**G**) Control panel for position tracking, defocus tracking and fitting.

The overview of the software user interface contains the seven sub-windows shown in Fig. 6: i) windows show the three tilting images from the current (Fig. 6A), previous (Fig. 6B) and next previous tilt angles (Fig. 6C) respectively; ii) window (Fig. 6D) shows the historical records of the X and Y errors and the defocus error; iii) window (Fig. 6E) shows the real-time FFT (or sub-area FFT) of the current tilting image against its fitted CTF (or targeted CTF); iv) window (Fig. 6F) is the tomographic control panel, which includes options for basic parameter selection, including the file directory, tilting angle range and step, tracking options, and progress bar. The bottom of this window (Fig. 6F) also contains a set of 5 buttons: start, view, acquire, next and run. These 5 buttons allow the user to switch between manual and automated data collection (see the below procedure for details). Moreover, window (Fig. 6A) contains an option to redefine the target center through the user mouse selection of the target object on this image, and window (Fig. 6E) contains an option to switch the display between real-time FFT and sub-area FFT (left panel) and between fitted CTF rings and expected CTF rings (right-panel) by clicking on the corresponding panel. Button “A” in window (Fig. 6F) contains even more advanced options for positional tracking and defocus tracking for high-level users (Fig. 6G).

The procedure to operate the tomography software is briefly explained as follows: i) move an interesting region to the image center by using the joystick; ii) input the data acquisition parameters in window (Fig. 6F); iii) click the “Start” button, which will automatically initialize the backlash, and then gradually move to the negative maximum starting angle; iv) click “Ctrl + mouse left button” on window (Fig. 6A) to re-adjust the target center when necessary. Do not use the TEM joystick, because it will disrupt the backlash setup; v) set the acquisition defocus (optionally, the user may set multiple image acquisitions under a series of defocus values); vi) press “Run” for automated collection (alternatively, for manual collection, press “Acquire” followed by “Next”); vii) monitor the data collection process (optionally, press the “Shift” key to pause data acquisition so the user can precisely redefine the center via Ctrl + mouse left button, as in step 4); viii) the program will automatically stop after the acquisition process is finished (similarly, the “Stop” button allows the user to halt the data acquisition process at any tilting angle).

## Example application

**Figure 7:**
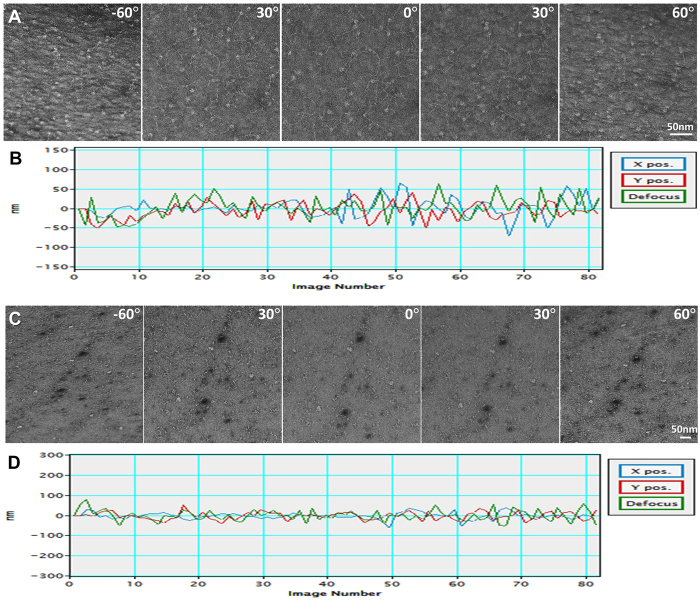
Automatic tomography data collection of negative-stained nucleosome-DNA complex and antibody conjugate. (**A**) A negatively stained nucleosome-DNA sample collected from −60° to +60° with a 1.5° step under 160,000× magnification and an expected defocus of 400 nm. (**B**) This series took 1.5 h to collect, with X/Y mean absolute positional errors and a defocus error of 17.8, 17.6, and 21.6 nm, respectively, with standard deviations of 24.6, 21.1 and 26.9 nm, respectively. (**C**) A negatively stained antibody conjugate sample collected from −60° to +60° with a 1.5° step under 80,000× magnification and an expected defocus of 800 nm. (**D**) This series took 1.5 h to collect, with X/Y mean absolute positional errors and a defocus error of 13.1, 17.1, and 22.9 nm, respectively, with standard deviations of 18.3, 20.7 and 29.1 nm, respectively.

**Figure 8:**
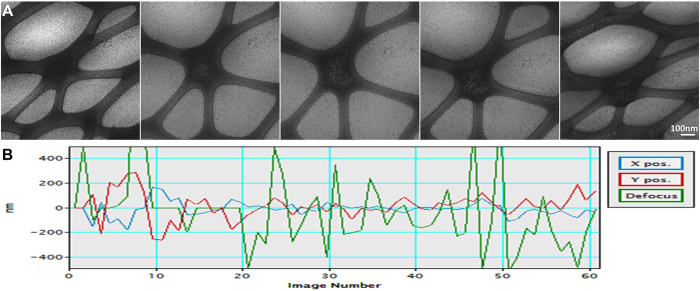
Automatic tomography data collection of low-density lipoprotein. (**A**) A Cryo-EM LDL sample collected from −60° to +58° with a 2° step under 50,000× magnification and an expected defocus of 2,000 nm. (**B**) This series took 1 h and 15 min to collect, with X/Y mean absolute positional errors and a defocus error of 43.0 nm 78.4, and 221.8 nm, respectively, with standard deviations of 61.1, 108.6 and 340.2 nm, respectively.

To validate the control capability of our automated ET, we tested this software on a Zeiss Libra 120 TEM and acquired the ET tilt series from three different samples: i) Negative-stained nucleosome-DNA complex. The tomographic data set was fully automatically acquired from −60° to +60° with a 1.5° step under a magnification of 160,000× and a target defocus of 400 nm (under focus, same as below) (Fig. 7A, Supporting Video 1). The acquisition took 1.5 h. The tracking of the acquisition processes showed that the means of the absolute errors of X, Y and defocus were 17.8, 17.6 and 21.6 nm, respectively (Fig. 7B), and the standard deviations (STD) were 24.6, 21.1 and 26.9 nm, respectively. The defocus error is within approximately 7% of the targeted defocus value. ii) Negative-stained antibody conjugate sample (The sample was generated by conjugating a small organic molecule to the free cysteine residues after the reduction of disulfide bonds of the antibody. It is expected that the sample would contain heterogeneous population of species due to the lack of disulfide bonds). The tomographic data set was fully automatically acquired from −60° to +60° with a 1.5° step under a magnification of 80,000× and a target defocus of 800 nm (Fig. 7C, Supporting Video 2). The acquisition took 1.5 h. The tracking of the acquisition processes showed that the means of the absolute errors of X, Y and defocus were 13.1, 17.1 and 22.9 nm, respectively, and the STDs were 18.3, 20.7 and 29.1 nm, respectively (Fig. 7D). The defocus error was within approximately 4% of the targeted defocus value. iii) Cryo-EM low-density lipoprotein (LDL) sample. The tomographic data set was fully automatically acquired from −60° to +58° with a 2° step under a magnification of 50,000× and an expected defocus of approximately 2 μm (Fig. 8A, Supporting Video 3). The acquisition took 1.25 h, with a total dose of 339 electrons per pixel, or approximately 60 e^−^/A^2^. The tracking showed that the means of the absolute errors of X, Y and defocus were 43.0, 78.4 and 221.8 nm, respectively, and the STDs of X, Y and the defocus error were 61.1, 108.6 and 340.2 nm, respectively (Fig. 8B). The defocus error was within approximately 20% of the targeted defocus value, which is significantly higher than those from the negative-staining samples but still useful. The above tests suggest that the error is sufficiently low, allowing for automatic tilt series acquisition without any human interruption.

## Discussion

Our fully mechanically controlled automated ET data acquisition program uses three key procedures. First, all XY motion control is conducted with backlash-corrected goniometer control, which greatly improves the positioning accuracy and resolves issues related to repeated beam tilt/shift alignment. Second, the PI control system and image feedback enable the system to dynamically track the position with low error. This process does not require additional images solely to track the alignment, because the control system relies on only historical data. Third, another PI control system integrates CTF fitting as the method to control the Z height for defocus feedback. These features were incorporated into a Gatan digital micrograph (DM)-based tomography software^23^, integrating both manual and automated data collection.

In our method, compared to other ET control software, such as UCSF Tomography^10^ and Serial EM^17^ (Table 1), the major benefits include keeping the pre-aligned/optimized beam conditions unchanged and achieving position tracking solely through mechanical (goniometer) control. However, the present automated ET methods generally apply beam tilting/shifting to control the position tracking, which could potentially destroy the perfect beam condition (through hysteresis and coherence problems) and degrade the image quality. Our backlash elimination method enabled a substantial improvement in the mechanical positional accuracy of the goniometer (by nearly 50 times for a Zeiss Libra 120 Plus TEM, as shown in Fig. 2B), ultimately attaining repeatable positional accuracy after backlash correction (for a Zeiss Libra 120 TEM, as shown in Fig. 2D). This allows for successful position tracking while keeping optimal beam conditions for high-quality imaging.

Moreover, the PI control strategy enables the system to dynamically perform position and defocus tracking in an unbiased manner (*i.e.,* regardless of being at high or low tilt angles, the tracking error is always around zero, as shown in Figs 5 and 6). However, the present pre-calibration or prediction methods^9^ have the risk of losing tracking due to larger trajectory errors, especially at high tilt angles or larger tilt steps.

Our closed-loop PI control system uses only historical data to track the position and defocus and therefore requires only one image to be collected at each tilt. This is particularly essential for some beam-sensitive samples, because it reduces unnecessary exposure. However, some of the present automated ET methods add further exposure through additional image acquisition to assist in determining the XY shift and defocus changes.

The limitations of our control software include i) the dependence of the system performance on accurate image feedback, meaning that entirely correct image feedback cannot be ensured, particularly for lower-dose images (this is of particular concern for cryo-EM samples, because areas with more carbon film can aid in contrast and tracking at the cost of a smaller target area). Unfortunately, the contrast of cryo-samples limited successful tracking under magnifications above 50,000×. ii) The system cannot reduce random error. iii) The trajectory error cannot be readily eliminated within the first few images, because the program must build up some historical data first (for samples with larger initial system errors, the user may need to manually click to redirect the control system back to the tracking area for the first few images).

In summary, our control strategy accurately tracks both XY and defocus through a fully mechanically automated ET data collection program. Our control strategy ensures that the optimized beam conditions and alignment remain unchanged during data acquisition, which is vital for higher-resolution imaging at intermediate to high magnification (50,000× for cryo samples to 160,000× for negative-stained samples). With these automated ET improvements, we hope to contribute to the efficiency and quality of ET technology toward the study of nanoscale biological objects.

### Program availability information

Software is available free-of-charge to academia end users (Berkeley Intellectual Property Case 2016-022).

## Additional Information

**How to cite this article**: Liu, J. *et al*. Fully Mechanically Controlled Automated Electron Microscopic Tomography. *Sci. Rep.* **6**, 29231; doi: 10.1038/srep29231 (2016).

## Supplementary Material

Supplementary Information

Supporting Video S1

Supporting Video S2

Supporting Video S3

## Figures and Tables

**Table 1:** Comparison of automated tomographic software packages.

Item	UCSF Tomography^10^	Serial EM^17^	Our software
TEM type	FEI, and JOEL	FEI, and JOEL	Zeiss Libra 120
Model-based goniometer tracking	Yes	No	No
XY tracking value	Pre-calibration of model parameter	Exploration method	On-line feedback control
XY tracking actuator	Beam tilt or shift	Beam tilt or shift	Goniometer
XY tracking limitation	Yes, by objective aperture	Yes, by objective aperture	No
XY tracking image feedback	Yes, cross-correlation	Yes, cross-correlation of filtered image	Yes, modified cross-correlation
XY tracking additional image	Yes, 2 low-mag. images overall	Yes, 2 high-mag. images overall	Yes, 2 high-mag. images overall
Defocus tracking value	On-line update on the basis of pre-calibrated parameters	Beam-tilt-induced image displacement method	On-line feedback control
Defocus tracking actuator	Change objective lens current	Beam tilt and shift; change objective lens current	Change objective lens current
Defocus tracking limitation	Yes, by an acceptable image quality	Yes, by an acceptable image quality	Yes, by an acceptable image quality
Z tracking image feedback	No, predicted by the XY shift	Yes, cross-correlation of the additional image	Yes, CTF fitting of historical images
Z tracking additional images	No	Yes, 2 additional images at each tilt	No
Tilt range	Two loops (0° to +60°; 0° to −60°)	One loop at lower mag. and two loops at higher mag.	One loop (−60° to +60°)
Optical backlash (hysteresis)	Yes	Yes	No
Mechanical backlash	No	No	No, eliminated by moving strategy
Optical rotation compensation	Off-line identification	Off-line identification	Off-line identification
Keep magnitude unchanged during collection process	No, go to low mag. Between two tilting loops	No, go to low mag. for tracking when mag. is larger than 50,000×	Yes

This table catalogs the basic ideas applied in our system compared with UCSF tomography and SerialEM software. Feature comparisons are listed with similarities and differences outlined.
