# Supplementary material for: Fully Mechanically Controlled Automated Electron Microscopic Tomography
Source: Sci Rep. 2016 Jul 11;6:29231. doi: 10.1038/srep29231 (PMC4941525; doi:10.1038/srep29231)
Supplement: Supplementary Information [file srep29231-s1.pdf]

**Fully Mechanically Controlled Automated Electron Microscopic Tomography**

Jinxin Liu, Hongchang Li, Lei Zhang, Matthew Rames, Meng Zhang, Yadong Yu, Bo Peng, César Díaz Celis, April Xu, Qin Zou, Xu Yang, Xuefeng Chen, Gang Ren

## Supporting Videos

**Supporting Video 1.** A representative electron tomographic tilt series (after alignment) of negative-stained DNA-nucleosome complex is acquired from −60° to +60° with a 1.5° step under a magnification of 160,000× and a target defocus of 400 nm.

**Supporting Video 2.** A representative electron tomographic tilt series (after alignment) of negative-stained antibody conjugate sample is acquired from −60° to +60° with a 1.5° step under a magnification of 80,000× and a target defocus of 800 nm.

**Supporting Video 3.** A representative electron tomographic tilt series (after alignment) of cryo-EM low-density lipoprotein (LDL) sample is acquired from −60° to +58° with a 2° step under a magnification of 50,000× and an expected defocus of approximately 2 μm.
